# Tibetan mineral-herbal medicine *Zuotai* alleviates the depressive-like behaviors in chronic restraint-stressed mice while regulating stress hormone, inflammation and monoamine

**DOI:** 10.3389/fphar.2023.1098378

**Published:** 2023-11-24

**Authors:** Cen Li, Cuiying Niu, Hongtao Bi, Jing Zhao, Jorge Ivan Alvarez, Farong Yuan, Xiangyun Gai, Lixin Wei, Yuzhi Du, Yajun Qiao, Hania Kebir, Yuancan Xiao, Hongxia Yang

**Affiliations:** ^1^ Qinghai Key Laboratory of Tibetan Medicine Pharmacology and Safety Evaluation, Northwest Institute of Plateau Biology, Chinese Academy of Sciences, Xining, Qinghai, China; ^2^ CAS Key Laboratory of Tibetan Medicine Research, Northwest Institute of Plateau Biology, Chinese Academy of Sciences, Xining, Qinghai, China; ^3^ Department of Pathobiology, School of Veterinary Medicine, University of Pennsylvania, Philadelphia, PA, United States; ^4^ Jinke Tibetan Medicine Co., Ltd., Xining, Qinghai, China; ^5^ School of Pharmacy, Qinghai Nationalities University, Xining, Qinghai, China

**Keywords:** Tibetan medicine, *Zuotai*, mercuric sulfide (HgS), depression, inflammation, monoamine neurotransmitters, brain-derived neurotrophic factor (BDNF), chronic restraint stress (CRS)

## Abstract

**Introduction:** *Zuotai* is an ancient mineral-herbal mixture containing β-HgS in Tibetan medicine. It is used to treat nervous system diseases, similar to Chinese medicine cinnabar and Indian Ayurveda medicine *Rasasindura*. However, one of the key problems faced by *Zuotai* is that its indications are ambiguous. Our previous study found that *Zuotai* exhibited the activity of ameliorating depressive-like behaviors in a chronic mild stress model. However, due to the inherent limitations of animal models in simulating human disease, clear results often require more than one model for confirmation.

**Methods:** Therefore, another depression model, chronic restraint stressed (CRS) mice, was used to validate the antidepression effect of *Zuotai*. Prophylactic treatment was conducted for 21 consecutive days while mice were subjected to chronic restraint stress.

**Results:** It was observed that *Zuotai* and β-HgS alleviated anhedonia, behavioral despair, stereotype behavior, and reduced exploratory and spontaneous movement in CRS mice. *Zuotai* and β-HgS also reversed the increases of stress hormone corticosterone (Cort) in serum and pro-inflammatory cytokines in serum and brain, and increased the serotonin in cortex in CRS mice, with positive dose-effect relationship. The number of Ki67-positive cells in the dentate gyrus and the level of brain-derived neurotrophic factor (BDNF) in the hippocampus were slightly elevated in CRS mice treated with *Zuotai*; however, there was no statistically significant difference. Although *Zuotai* increased the total Hg concentration in main organs, the levels remained below those needed to result in observed adverse effect, at least for kidney and liver; and *Zuotai* showed no observed adverse effect on the brain histopathology, the cell proliferation in dentate gyrus, as well as the hippocampal and cortical organ coefficients.

**Conclusion:** *Zuotai* exhibited the alleviation of depressive-like behaviors in CRS mice, accompanying with ameliorating stress hormone, peripherical and cerebral inflammation, and monoamine neurotransmitter.

## Graphical Abstract

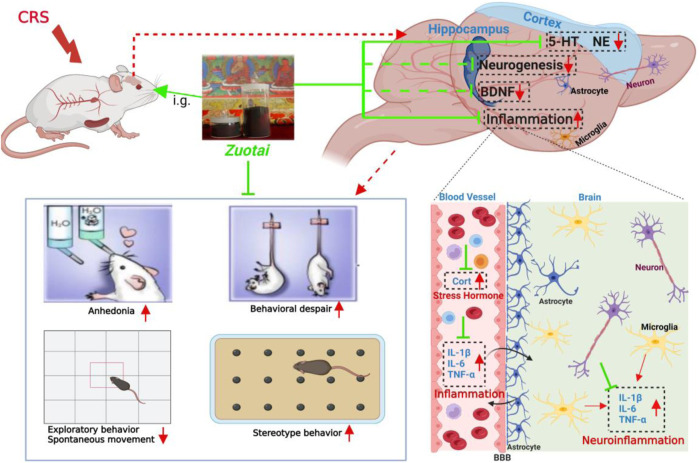

## 1 Introduction

Depression is a common global mental disorder, estimated to affect 3.8% of the population (World Health Organization, 2017). It has a serious impact on the patient’s daily life, work, study, and family interactions, and at its worst can lead to suicide (World Health Organization, 2017). Although great progress has been made in the treatment of depression, the remission and response rate of depression still need to be improved due to the high heterogeneity of depression and the complexity of the etiologies (Malhi and Mann, 2018). A clinical study by the National Institute of Mental Health found that even after treatment with first, second, third, and fourth acute treatment steps, the overall cumulative remission rate was only 69% (Rush et al., 2006). In addition, a systematic review of mid- and long-term outcomes reported that up to 80% of patients with treatment-resistant depression relapsed within a year of achieving symptom remissions (Fekadu et al., 2009). Therefore, there is still a need to develop new antidepressant therapies, including pharmacotherapies.

Traditional medicine is an importantly potential alternative for discovering new antidepressant drugs. Tibetan medicine is characterized by regional and easy accessibility, and affordability in the Qinghai-Tibet Plateau region. *Zuotai* is a classic mineral-herbal mixture containing about 54% mercuric sulfide (HgS) in Tibetan medicine. It was first recorded in the book *“Four Medical Classics”* in the 8th century and is called by Tibetan medical practitioners as the “King of Nectar Essence”. *Zuotai* is commonly used as a key component in many compound preparations for treating nervous system diseases (Yan et al., 2007; Wang, 2010; Li et al., 2015a), and some its compound preparations (such as *Qishiwei Zhenzhu Wan, Renqing Changjue, Renqing Mangjue, et.al.*) are included in Chinese Pharmacopoeia (2020 Version). In the Indian Ayurvedic medicine system, mercuric sulfide drugs (such as *Rasasindura*) are often used to treat nervous disorders, insomnia, and tongue hemiplegia (Kamath et al., 2012; Liu et al., 2019). According to the Chinese Pharmacopoeia, cinnabar (α-HgS ≥96%) improves moods, soothes nerves, and the improves eyesight, and its comound preparations are clinically used to treat insomnia, low mood, restlessness, palpitations, and epilepsy (Pharmacopeia Committee, 2015; Liu et al., 2018). Moreover, animal studies showed that cinnabar significantly alleviated the behavioral despair state of mice, using the forced swimming and tail suspension tests, increased the distance of movement in the open field test (Dai et al., 2014), and altered brain tissue serotonin (5-HT) levels (Wang et al., 2007). Our latest study showed that metacinnabar (β-HgS) enhanced the effects of antidepressants in mice (Qiao et al., 2022). Moreover, clinical studies have reported that *Zhusha Anshen Wan*, a cinnabar compound preparation, ameliorated the symptoms of patients with depression (Dong, 2008; Wu, 2015). We therefore hypothesized that HgS-containing *Zuotai* had a similar effect as cinnabar in relieving depressive symptoms.

Advances in the pathophysiological understanding of depressive disorder have revealed the multi-faceted neuro-biomechanical features of this disorder. The monoamine neurotransmitter hypothesis states that depression is caused by the functional deficiency of monoamine neurotransmitters in the central nervous system, so almost all clinical antidepressant drugs used so far have been developed based on this hypothesis (Cosci and Chouinard, 2019). The neuroendocrine hypothesis states that the hypothalamic-pituitary-adrenal (HPA) axis is closely related to the occurrence and development of depression, and it has been clinically found that patients with depression had hyperfunction of the HPA axis, with antidepressant treatment restoring it to normal levels (Pariante and Lightman, 2008). The important roles of peripheral inflammatory responses and neuroinflammation in the pathogenesis of depression have also been characterized and confirmed, such as elevated pro-inflammatory cytokines in peripheral serum and cerebrospinal fluid, and activated microglia in the brain of patients with clinical depression. A similar inflammatory activation has been found in animal models of chronic stress, and inflammatory stimuli also induced depression-like behaviors in animals (Remus and Dantzer, 2016). In addition, depression was often accompanied by abnormal neuroplasticity that occurred in the prefrontal cortex, hippocampus, amygdala, and other limbic systems (Patel et al., 2019). Furthermore, there is a strong correlation between the antidepressant effects of antidepressants and neuroplastic responses (Yang et al., 2020). The role of neuronal plasticity, at least in part, is mediated by brain-derived neurotrophic factor (BDNF) signaling (Yang et al., 2020). Our previous study found that *Zuotai* had antidepressant activity in chronic unpredictable mild stress (CUMS) mice, and was involved in the regulation of the HPA axis and monoamine neurotransmitters (Zhao et al., 2018). There is a consensus that each animal model has more or less inherent limitations in terms of face validity, construct validity, and predictive validity, and almost any single animal model cannot fully simulate the characteristics of human depression (Söderlund and Lindskog, 2018; Planchez et al., 2019a). Therefore, it often requires more than one model for validation, and it is still unclear whether the antidepressant activity of *Zuotai* also involves regulation of neuroinflammation and BDNF.

We therefore used another classic animal model of depression, the chronic restraint stress model, for further verification, and to characterize the alleviating effects of *Zuotai* and β-HgS on the mood-like behaviors of chronic restraint-stressed mice, including interventions on stress hormone, inflammation, BDNF, monoamine neurotransmitters, and hippocampal histology. In addition, mercury is a naturally occurring heavy metal that is ubiquitous in the environment. Although mercuric sulfide used in traditional medicine is insoluble (*Ksp* of *β*-HgS is 1.6 × 10^−52^; *Ksp* of *α*-HgS is 4.0 × 10^−53^) (Dean, 1999) and is considered by EPA to be very difficult to be absorbed by gastrointestinal tract (EPA, 2002), there is a need to be concerned about the potential risk of mercury exposure. So, in this study, we also determined the total Hg in the brain and other major organs of mice.

## 2 Materials and methods

### 2.1 *Zuotai*

*Zuotai* (No. 20110705, Tibetan Pharmaceutical Factory, Tibet Autonomous Region, China).

Generally, *Zuotai* contains around 54% HgS and 40% elemental sulfur (S_8_), as well as small amounts of other substances such as minerals and organic matters (Yan, 2007; Yan et al., 2007; Wang et al., 2010b; Xia et al., 2010; Yan and Ma, 2010; Zhao et al., 2013; Li et al., 2015b; Li et al., 2015a; Li et al., 2016; Li et al., 2021; Zhang et al., 2018). *Zuotai* is a black ultra-fine powder preparation obtained by special processing technology from elemental sulfur (S_8_), elemental mercury (Hg^0^), eight-metals (gold, silver, red copper, brass, bronze, iron, zinc, lead) ash, and eight-minerals (gold ore, silver ore, limestone (*Quan Shi*), magnetite, pyrite, realgar, orpiment, biotite) ash with a mass ratio of 50, 50, 0.333, and 0.333 (Gama et al., 1990; Xia, 2010). Before grinding mercury (Hg^0^) and sulfur (S_8_)together, it is necessary to wash them multiple times with the solutions of plant-based auxiliary materials to remove the impurities and purify mercury (Hg^0^) and sulfur (S_8_). These plant-based auxiliary materials include Rhizoma alpinae officinarum (Alpinia officinarum Hance), garlic (Allium sativum L.), black pepper (Piper nigrum L.), biota seed (Fructus piperis Longi), seabuckthorn (Hippophae rhamnoides L.) paste, highland barley sour wine, Fructus terminaliae Billericae, Fruit of medicine Terminalia (Terminalia chebula Retz.), rapeseed oil, Acorus gramineus, emblic (Fructus phyllanthi), Herba dinathi, Apocynum venetum L., Aconitum pulchellum, Lalang grass Rhizome, castor oil, croton oil, black sesame oil, and pomegranate seed decoction. Then, equal quantities of cleaned mercury and sulfur are put together (here excess sulfur ensures the full reaction of elemental mercury), and grind them thoroughly to obtain a black powdered reaction product. 100 parts of the aforementioned reaction product, add 0.333 parts of eight-metals ash and 0.333 parts of eight-minerals ash to obtain a final mixture called *Zuotai* (Gama et al., 1990; Xia, 2010).

**FIGURE 1:**
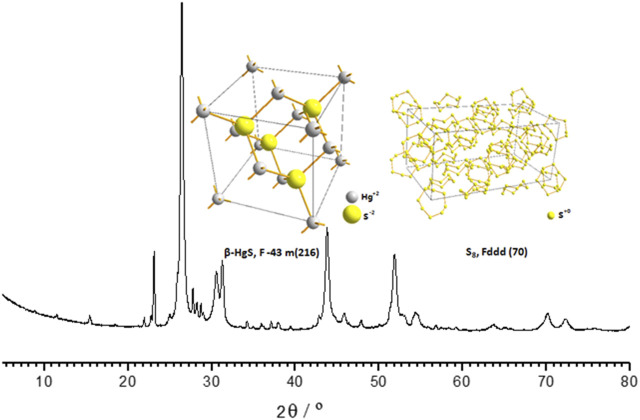
XRD spectrum of *Zuotai* and its major components.

The *Zuotai* sample used in this study was determined by X-ray diffractometers (XRD, X’ per Pro, PANalytical, Almelo, Netherlands) and automatic direct mercury analyzer (DMA-80, Milestone S.r.l., Milan, Italy). We found that the main components in this *Zuotai* sample were cubic crystal mercuric sulfide (β-HgS) and elemental sulfur (S_8_) as shown in Figure 1 (Li et al., 2015a). Based the chemical form of Hg in *Zuotai*, here we further calculated the percentage of β-HgS in *Zuotai* is 54.0928% ± 0.1823% (n = 3) after assaying total Hg by using automatic direct mercury analyzer, which is consistent with the results reported in previous reports (Wang et al., 2010b; Xia et al., 2010; Zhang et al., 2018; Li et al., 2021). The result is shown in Table 1.

**TABLE 1:** β-HgS percentage in *Zuotai* (n = 3, mean ± SD).

No.	*Zuotai*, g	Total Hg, g	β-HgS weight calculated, g	Total Hg percentage in *Zuotai*, %	β-HgS percentage in *Zuotai*, %
repeat 1	0.2093	0.09807	0.1137	46.8573	54.3476
repeat 2	0.2029	0.09447	0.1096	46.5578	54.0002
repeat 3	0.2077	0.09658	0.1120	46.4979	53.9307
Mean	–	–	–	46.6376 ± 0.1572	54.0928 ± 0.1823

### 2.2 Reagents

β-HgS (LOT: 1344-48-5, 98% mercury sulfide content; Alfa Aesar, Haverhill, MA, USA). Soluble starch (AR, Sinopharm Chemical, Shanghai, China), imipramine hydrochloride (113-52-0, 98%; 3B Scientific, Hamburg, Germany), sucrose (AR, Tianjin Beichen Fangzheng Reagent Factory, Tianjian, China), paraformaldehyde (AR, Tianjin Guangfu Institute of Fine Chemical Industry, Tianjin, China), mouse IL-6 ELISA kits (85-BMS603/2; eBioscience, CA, USA), mouse IL-6 (ELM-IL6-1, Raybiotech, GA, USA), mouse IL-1β ELISA kits (85-BMS6002; eBioscience, CA, USA), mouse IL-1β (ELM-IL1b-1, Raybiotech, GA, USA), mouse TNF-α ELISA kits (85-BMS607/3, eBioscience, CA, USA), and mouse TNF-α (ELM-TNFa-1, Raybiotech, GA, USA), norepinephrine enzyme ELISA kits (CEA907Ge; Cloud-Clone, Wuhan, China), serotonin ELISA kits (CEA808Ge; Cloud-Clone, Wuhan, China), brain-derived neurotrophic factor (BDNF) ELISA kits (SEA011Mu; Cloud-Clone, Wuhan, China), Corticosterone (Cort) ELISA kits (CSB-E07969m, CUSABIO, Wuhan China), total mercury standard solution (1 mg/mL, GSB04-1729-2004; National Nonferrous Metals and Electronic Materials Analysis and Testing Center, China), nitric acid (GR, Baiyin Liangyou Chemical Reagent Company, Gansu, China), high purity oxygen (purity ≥99.99%, Xining Laoqian Gas Trading Company, China).

### 2.3 Animals

SPF grade KM male mice were purchased from the Experimental Animal Center of Gansu University of Chinese Medicine [License number: SCXK (Gan) 2015-0002] for animal experiment 1 and 2. SPF grade KM male mice were purchased from the Beijing Vital River Laboratory Animal Technology Co., Ltd. [License number: SCXK (Jing)2021-0006] for animal experiment 3. Feed and wood shaving bedding were purchased from Beijing Keao Xieli Feed Company (Beijing, China). The drinking water was sterilized. All experimental animals were fed and housed in the SPF animal facility at the Northwest Plateau Institute of Biology of the Chinese Academy of Sciences, with a temperature of 20–26°C, humidity of 40–70%, and mechanical ventilation and artificial lighting involving a 12-h day–night cycle. Animals had free access to food and drinking water. The study was approved by the Ethical Review Committee of Laboratory Animals of the Northwest Institute of Plateau Biology of Chinese Academy of Sciences (No. NWIPB20160325-01), with strict adherence with the National Institutes of Health Guide for the Care and Use of Laboratory Animals (NIH Publication No. 8023, revised in 1978). Animals were euthanized after the experiments were completed.

### 2.4 Equipment

Equipment involved an ultrapure water instrument (Milli-Q Reference; Millipore, Burlington, MA, USA), homemade restraint tubes (50 mL centrifuge tubes, 3–4 0.2 cm holes in each tube); rat and mouse open-field activity system (OFT-100; Chengdu Taimeng Software), homemade tail-suspension experimental device, hard disk video camera (GZ-MG174AC; JVC, Yokohama, Japan), white glass beads (1.5 cm in diameter), semi-automatic paraffin microtome (LS-2055; Shenyang Shoulong Electronic Instrument Company), pathological imaging system (DSC-H50; Sony, Tokyo, Japan), high-throughput tissue grinder (Scientz-48; Ningbo Scientz Biotechnology Co., Ltd., Ningbo, China), highspeed freezing centrifuge (Sigma 3K-15; Sigma-Aldrich Company), multifunctional microplate reader (Enspire2300; Perkin Elmer, Waltham, MA, USA), 1/10,000 electronic balance (ME204: Mettler Toledo, Oakland, CA, USA), plate shaker, micropipetters (20, 100, 200, and 1,000 μL; Thermo Fisher Scientific, Waltham, MA, USA), pH meter (PB10; Sartorious, Gottingen, Germany), stopwatch timer (Shanghai Stopwatch, Shanghai, China), and automatic direct mercury analyzer (DMA-80, Milestone S.r.l., Milan, Italy).

### 2.5 Animal grouping and administration

#### 2.5.1 Animal experiment 1

**FIGURE 2:**
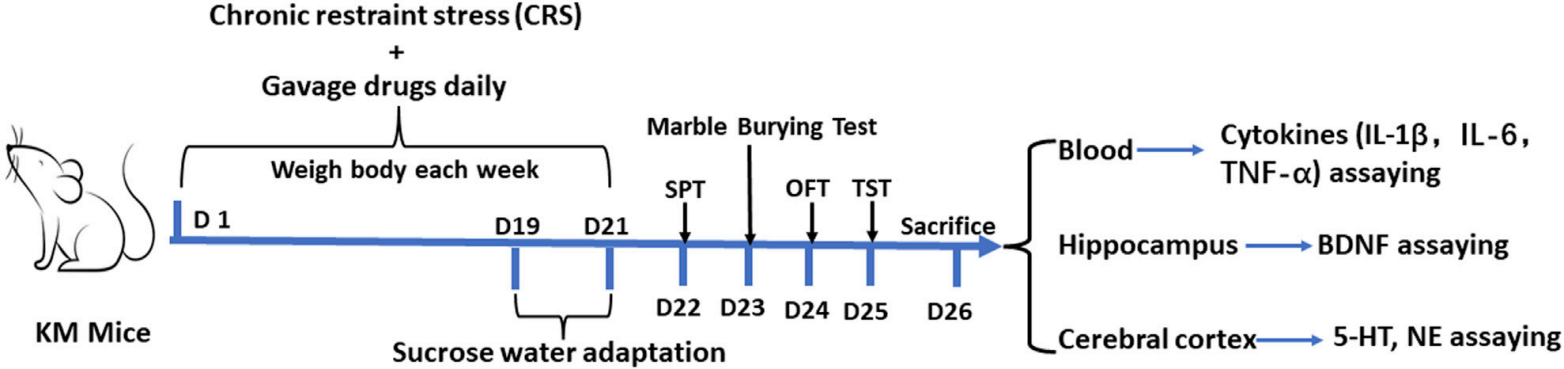
Processing flowchart of animal experiment 1. Abbreviations: SPT (Sucrose preference test), TST (Tail suspension test), OFT (Open field test), D (Day), IL-1β (Interleukin 1 beta), IL-6 (Interleukin 6), TNF-α (Tumor necrosis factor alpha), BDNF (Brain-derived neurotrophic factor), 5-HT (Serotonin), and NE (Norepinephrine).

The KM mice were fed in the SPF facility for 7 days to adapt the new environment, and were randomly divided into seven groups with 10 mice in each group according to their body weight and the behavior results of the open field test. The seven groups included the control group, chronic restraint stress model group (CRS + Vehicle) group, chronic restraint stress (CRS)+imipramine (IMI, an anti-depressant) (positive control, 15 mg/kg) group, the CRS + *Zuotai* low dose group (CRS + *Zuotai* L), the CRS + *Zuotai* medium-dose group (CRS + *Zuotai* M), the CRS + *Zuotai* high dose group (CRS + *Zuotai* H), and the CRS+β-HgS group (CRS+β-HgS) group. Model establishment and drug administration were performed simultaneously, namely prophylactic treatment. The dosage used in each group is shown in Table 2. The animals were administered intragastrically once a day, the body weight was measured every 7 days, and the administration volume was adjusted according to the body weight. The animals were subjected to the sucrose preference test, marble burying test, open field test, and tail suspension test on the 22nd, 23rd, 24th, and 25th day, after the above treatments, respectively. On the 26th day, the animals were anesthetized using pentobarbital (0.3%, 50 mg/kg body weight). Blood samples were collected from the orbital venous plexus assaying cytokines (IL-1β, IL-6, and TNF-α). After euthanasia, organ tissue samples such as the hippocampus and cerebral cortex were also collected and stored at −20°C for assaying. The detailed processing flow is shown in Figure 2.

**TABLE 2:** Animal grouping and administration.

Groups	Chronic restraint stress (CRS)	Doses of drugs mg/kg·day	Dose of pure HgS in drugs mg/kg·day	Duration days
Control	-	0.2% gelatinized starch solution	0	21
CRS + Vehicle	+	0.2% gelatinized starch solution	0	21
CRS + IMI	+	15 (imipramine), in 2% gelatinized starch solution	0	21
CRS + *Zuotai* L	+	6.070 (*Zuotai*), in 2% gelatinized starch solution	3.3153	21
CRS + *Zuotai* M	+	60.70 (*Zuotai*), in 2% gelatinized starch solution	33.153	21
CRS + *Zuotai* H	+	607.0 (*Zuotai*), in 2% gelatinized starch solution	331.53	21
CRS+β-HgS	+	3.383 (β-HgS), in 2% gelatinized starch solution	3.3153	21

Note: (1) The setting of dosage. The human dose of *Zuotai* is 0.667 mg/kg/day, and the equivalent dose factor of mouse to human is 9.3, so we used 6.070 mg/kg/day as the low dose for the mice in the CRS + *Zuotai* L group. And, in order to explore whether *Zuotai* has a dose-dependent effect on chronic restraint stress mice and whether *Zuotai* poses an observed Hg exposure risk at very high doses for long-term administration, so we set another two doses for *Zuotai*, 66.7 mg/kg/day (10 times dose of CRS + *Zuotai* L) for CRS + *Zuotai* M group and 667.0 mg/kg/day (100 times dose of CRS + *Zuotai* L) for CRS + *Zuotai* H group. Besides, our previous study found that Hg in the *Zuotai* was in the form of insoluble HgS, with an average content of 54.5%, representing a total Hg content of 46.98% (Wang et al., 2010a; Xia et al., 2010). The purity of commercial β-HgS (1344-48-5; Alfa Aesar), used in this study was 98%. We set the β-HgS dose in the CRS+β-HgS group as 3.383 mg/kg/day, namely the pure β-HgS dose is 3.3153 mg/kg/day. So, the pure β-HgS dose in the CRS+β-HgS group was similar to that (pure β-HgS dose: 3.3153 mg/kg/day) in the CRS + *Zuotai* L group. (2) The purpose of using 2% gelatinized starch solution to suspend drugs. Due to the fact that *Zuotai* (mainly composed of insoluble mercury sulfide and sulfur) and β-HgS are heavy and extremely fine powder here, both of them need to be evenly suspended with a 2% gelatinized starch solution to ensure the accuracy of the dosage of *Zuotai and* β-HgS when they were administered to mice by gavage.

#### 2.5.2 Animal experiment 2

**FIGURE 3:**
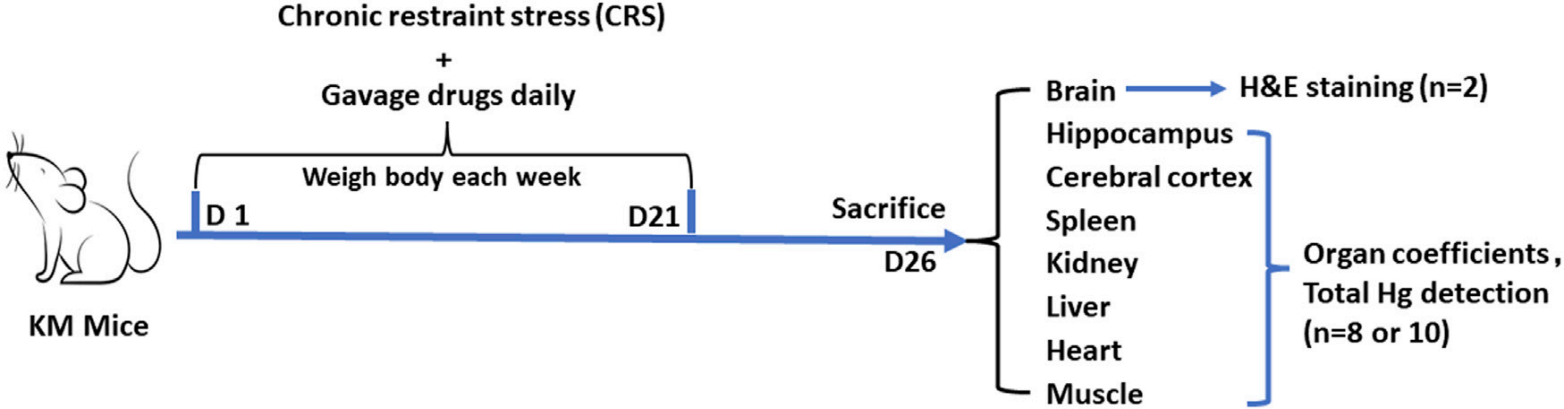
Processing flowchart of animal experiment 2. Abbreviations: H&E, Hematoxylin and eosin; D, Day.

Animal experiment 2 was conducted to detect the total mercury contents in organs and observe the pathological changes of the hippocampus to assess the risk of mercury accumulation in the CRS mice treated with *Zuotai* and cubic crystal system mercuric sulfide (β-HgS). The grouping (control group, CRS + Vehicle group, CRS+ IMI group, CRS + *Zuotai* L, CRS + *Zuotai* M, CRS + *Zuotai* H, and CRS+β-HgS group), chronic restraint stress, and drug administration were the same as previously mentioned in Table 2. 10 mice were in each group. Model establishment and drug administration were performed simultaneously, namely prophylactic treatment. At the 26th day, pentobarbital (0.3%, 50 mg/kg body weight) was intraperitoneally injected for anesthesia. Blood was collected from the orbital venous plexus. Whole brain tissues of 2 mice in each group were collected and fixed with 4% paraformaldehyde. In addition, tissue samples of other organs in each group, such as the hippocampus (n = 8), cerebral cortex (n = 8), and other organs (kidney, liver, spleen, heart, and hind leg muscles, n = 10) were collected for total mercury detection. The detailed processing is shown in Figure 3.

#### 2.5.3 Animal experiment 3

**FIGURE 4:**
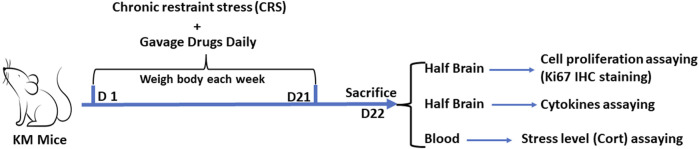
Processing flowchart of animal experiment 3. Abbreviations: IHC, Immunohistochemistry; Cort, Corticosterone; D, Day.

Animal experiment 3 was conducted to assess the effect of *Zuotai* and β-HgS on the stress level in CRS mice serum and the cell proliferation and inflammation level in the brains of CRS mice. The grouping (control group, CRS + Vehicle group, CRS+ IMI group, CRS + *Zuotai* L group, CRS + *Zuotai* M group, CRS + *Zuotai* H group, and CRS+β-HgS group), chronic restraint stress, and drug administration were the same as previously mentioned in Table 2. Eight mice were in each group. Model establishment and drug administration were performed simultaneously, namely prophylactic treatment. At the 22nd day, mice were intraperitoneally injected with pentobarbital (0.3%, 50 mg/kg body weight) for anesthesia. Blood was collected from the orbital venous plexus for assaying the stress level (serum Corticosterone). Half-brain (longitudinally equal cut) of each mouse was collected and fixed with 4% paraformaldehyde for assess the cell proliferation (Ki67 IHC staining) in dentate gyrus, and the another half-brain was collected and stored in −20°C for assaying cytokines (IL-1β, IL-6, and TNF-α). The detailed processing is shown in Figure 4.

#### 2.5.4 Preparation of solutions and suspensions

(1) The preparation of 2% gelatinized starch solution

4 g edible starch was added to 200 mL of ultrapure water, boiled, and cooled.

(2) The preparation of three *Zuotai* suspensions and β-HgS suspension

Due to *Zuotai* and β-HgS are insoluble in most of solvents except aqua regia or dissolvable sulfide solutions, it was necessary to use 2% gelatinized starch solution to suspend them. And, the volume of gavage was 10 mL solution/kg bw for mouse. Therefore, three concentrations (0.607, 6.070, and 60.70 mg/mL) of *Zuotai* suspensions and the 0.3383 mg/mL β-HgS suspension were prepared by suspending *Zuotai* powder or β-HgS powder in 2% gelatinized starch solution for CRS + *Zuotai* L group (6.070 mg/kg bw), CRS + *Zuotai* M group (60.70 mg/kg bw), CRS + *Zuotai* H group (607.0 mg/kg bw), and CRS+β-HgS group (3.383 mg/kg bw), respectively.

(3) The preparation of imipramine in 2% gelatinized starch solution

Imipramine is easily soluble in water. And the volume of gavage was 10 mL/kg bw for mouse. So, 1.5 mg/mL imipramine solution was prepared by solving imipramine in 2% gelatinized starch solution for CRS+IMI group (15 mg/kg bw).

#### 2.5.5 Establishment of the CRS mouse model

The chronic restraint stress (CRS) mouse model was established as previously reported (Kim and Han, 2006; Chiba et al., 2012), with slight adjustments. Specifically, the mice were placed in homemade restraint tubes, which were made from 50 mL centrifugation tubes, with 3–4 holes in the tube wall, to ensure good ventilation. The mice moved within the limited space in the restraint tube without causing visible physical harm. The mice were restrained for 6 h every day (10:00–16:00) and continued for 21 days.

### 2.6 Behavioral tests

#### 2.6.1 Sucrose preference test (SPT)

On the 19th day of the experiment, sucrose adaptation training was conducted according to a previously reported method (Dang et al., 2009). 72 h before the sucrose preference test, two water bottles were placed in each cage at the same time, one was 1% sucrose water solution, and the other was pure water. The positions of the sucrose water and pure water bottle were moved every 12 h to avoid the effect of positional preference. After 48 h of training, the mice were fasted for 22 h. The sucrose preference test was performed at 19:00 on the 22nd day. One bottle of 1% sucrose water and one bottle of pure water were placed in each cage, and the mice were allowed to drink water freely for 2 h. The consumption of sucrose water and pure water was measured. The sucrose preference rate (%) = [sucrose solution consumption/(sucrose solution consumption + pure water consumption)] × 100%.

#### 2.6.2 Marble burying test

The marble burying test was performed at 19:00 on the 23rd day, based on the method reported by Deacon et al. (2009). Before the experiment, plastic boxes (22 cm × 10 cm × 15 cm, containing 3 cm thick bedding) were prepared. Twenty-four glass beads were placed on the surface of the bedding parallel and evenly in each box. The mice were placed in the boxes and removed after 30 min. The number of glass beads buried by the mice in each group was recorded. Beads were reported as covered if two-thirds or more of the total volume was buried.

#### 2.6.3 Open field test (OFT)

The open field test was conducted at 19:00 on the 24th day in a quiet environment according to the method of Choleris et al. (2001). The test was used to evaluate the autonomous activity state and spatial exploration ability of mice. The mice were moved to the testing room 1 h in advance to reduce stress. A nine-square division model was chosen. The proportion of the central area was 0.5, and the standing area was adjusted. The size of the bottom was 500 × 500 mm. Non-experimental mice were used to debug the software to work normally. The experimental mice were removed from their cages and placed in the center of the apparatus, and the experiment was conducted in an appropriate brightness environment. The software automatically recorded the activity of the mice in the open field for 5 min. After each test, the mice were placed back into their cages, and mouse feces and urine left in the previous test were thoroughly cleaned, and then wiped with 75% ethanol and dried with paper towels.

#### 2.6.4 Tail suspension test

The tail suspension test was conducted at 19:00 on the 25th day according to the method reported by Steru et al. (1985). Specifically, a strip of adhesive tape was wrapped at 1–2 cm from the tip of the mouse tail, and then it was fixed on a wooden board with adhesive tape to ensure the tail of the hung mouse was vertical and the abdomen of the mouse was facing the camera. A high-definition digital video camera was used to record the behavior of the mice for 6 min. After all experiments were completed, an interval observation method was used to divide the video of the last 4 min into 5 s small segments to record the time when the mice were suspended and completely motionless.

### 2.7 The tests of Cort, monoamine transmitters, pro-inflammatory cytokines, and BDNF

Before Elisa assaying, the serum was acquired by centrifuging mouse whole blood at 3,000 rpm for 10 min. The cerebral cortex, hippocampus and half-brain were homogenized in a homogenizer (Scientz-48, Ningbo Scientz Biotechnology Co., Ltd., Ningbo, China; frequency: 60Hz, speed: 1800 times/min, 2 min) after adding the tissue lysis buffer (IS007, Cloud-Clone Corp., Wuhan, China), and then the supernatant was obtained by centrifugation (10,000 rpm, 5 min). The Elisa method was used to determine the corticosterone (Cort, CSB-E07969m, CUSABIO, Wuhan China), IL-1β (85-BMS6002, eBioscience, CA, USA), IL-6 (85-BMS603/2, eBioscience, CA, USA) and TNF-α (85-BMS607/3, eBioscience, CA, USA) in mouse serum, the 5-HT (CEA808Ge; Cloud-Clone, Wuhan, China) and norepinephrine (NE, CEA907Ge; Cloud-Clone, Wuhan, China) in the cerebral cortex, the brain-derived neurotrophic factor (BDNF, SEA011Mu; Cloud-Clone, Wuhan, China) in the hippocampus, and the IL-1β (ELM-IL1b-1, Raybiotech, GA, USA), IL-6 (ELM-IL6-1, Raybiotech, GA, USA) and TNF-α (ELM-TNFa-1, Raybiotech, GA, USA) in mouse brain. The detailed procedures were followed the instructions of the corresponding ELISA kits.

### 2.8 H&E staining

Two mice were selected from each group in animal experiment 2 at the 26th day, and whole brain tissues were collected, fixed with 4% paraformaldehyde for 24 h at room temperature, followed by tissue dehydration, transparency, embedding, sectioning, and dewaxing. For hematoxylin staining: slides were put into hematoxylin dye solution for 3 min, rinsed with tap water, differentiated with differentiation solution, rinsed with tap water, counterstained with the counterstaining solution, and rinsed with running water. For eosin staining: sequentially placed the slices into 85% and 95% gradient alcohol for 5 min each for dehydration, and stained in eosin staining solution for 5 min. For dehydration and sealing: sequentially placed the slices into anhydrous ethanol I for 5 min, anhydrous ethanol II for 5 min, anhydrous ethanol III for 5 min, dimethylbenzene I for 5 min, and dimethylbenzene II for 5 min until transparent, and sealed with neutral gum. Then an optical microscope was used for image acquisition and analysis.

### 2.9 Immunohistochemistry (IHC) staining

The four mice were selected from each group in animal experiment 3 at the 22nd day, and the half-whole brains were collected, fixed with 4% paraformaldehyde for 24 h at room temperature, followed by tissue dehydration, transparency methods, embedding, sectioning, and dewaxing. The Ki67 IHC staining was conducted as following steps. Antigen retrieval: slides were submerged in preheated citrate-EDTA antigen retrieval solution (95°C) for 30 min; after natural cooling, placed the slides in 1 X PBS (PH 7.4) for washing 3 times, 5 min each time. Inhibition of endogenous peroxidase: placed the slices in a 3% hydrogen peroxide solution, and incubated at room temperature in the dark for 25 min; placed the slides in 1X PBS (PH 7.4) for washing 3 times, 5 min each time. Serum blocking: dropped 3% donkey serum evenly over the tissue in the histological circle, and blocked at room temperature for 30 min. Primary antibody incubation: gently removed the blocking solution, dropped the rabbit anti- Ki67 antibody (ab15580, Abcam, Cambridge, UK; 1:500 in 1X PBS) solution onto the tissues, and incubated the slide flat in a humid box at 4°C overnight. Secondary antibody incubation: washed slides in 1X PBS (PH7.4) for 3 times, 5 min each time; then dropped the goat anti-rabbit secondary antibody labeled with HRP (GB23303, Servicebio, Wuhan, China; 1:200 in 1X PBS) into the histological circle to cover the tissue, and incubated at room temperature for 50 min. DAB staining: washed slides in 1X PBS (PH7.4) for 3 times, 5 min each time; after slightly shaking off excess liquid, dropped freshly prepared DAB staining solution into the histological circle; control the staining time under a microscope, positive results appeared as brownish-yellow, then rinsed the slide with tap water to stop staining. Counterstaining of cell nuclei: counterstained with hematoxylin for about 3 min, rinsed with tap water, differentiated with differentiation solution for a few seconds, rinsed with tap water, and blued with bluing reagent. Rinsed with running water. Dehydration and sealing: dehydrated and made transparent by sequentially placing the slide in 75% ethanol for 5 min, 85% ethanol for 5 min, anhydrous ethanol I for 5 min, anhydrous ethanol II for 5 min, n-butanol for 5 min, xylene I for 5 min. After dehydration, took out the slide from xylene and let it dry slightly before sealing it with mounting medium. Then an optical microscope was used for image acquisition and analysis.

### 2.10 Organ coefficient

After blood samples were collected on the 26th day, the hippocampus, cerebral cortex, spleen, kidney, heart, and other organs were quickly isolated. Blood on the isolated organs was washed with normal saline, and the residual water was absorbed with filter paper. The organ weight was measured using a balance. The organ coefficient calculation formula = organ weight (mg)/mouse body weight (g).

### 2.11 Determination of total mercury content

Mercury contents in the hippocampus, cerebral cortex, spleen, kidney, heart, liver, muscle, and other organ samples collected on the 26th day were measured using an automatic direct mercury analyzer (DMA-80, Milestone S.r.l., Milan, Italy) to evaluate the risk of mercury accumulation from *Zuotai* and β-HgS treatments.

### 2.12 Statistical methods

The data were expressed as the Mean ± SEM, and GraphPad 8 software (GraphPad, San Diego, CA, USA) was used for graphing and statistical analysis. One-way analysis of variance was used for multiple group comparisons was used to determine whether the data conformed to a normal distribution. *p* < 0.05 was considered statistically significant.

## 3 Results

### 3.1 *Zuotai* alleviated anhedonia, behavioral despair, and repetitive stereotyped behavior of CRS mice

**FIGURE 5:**
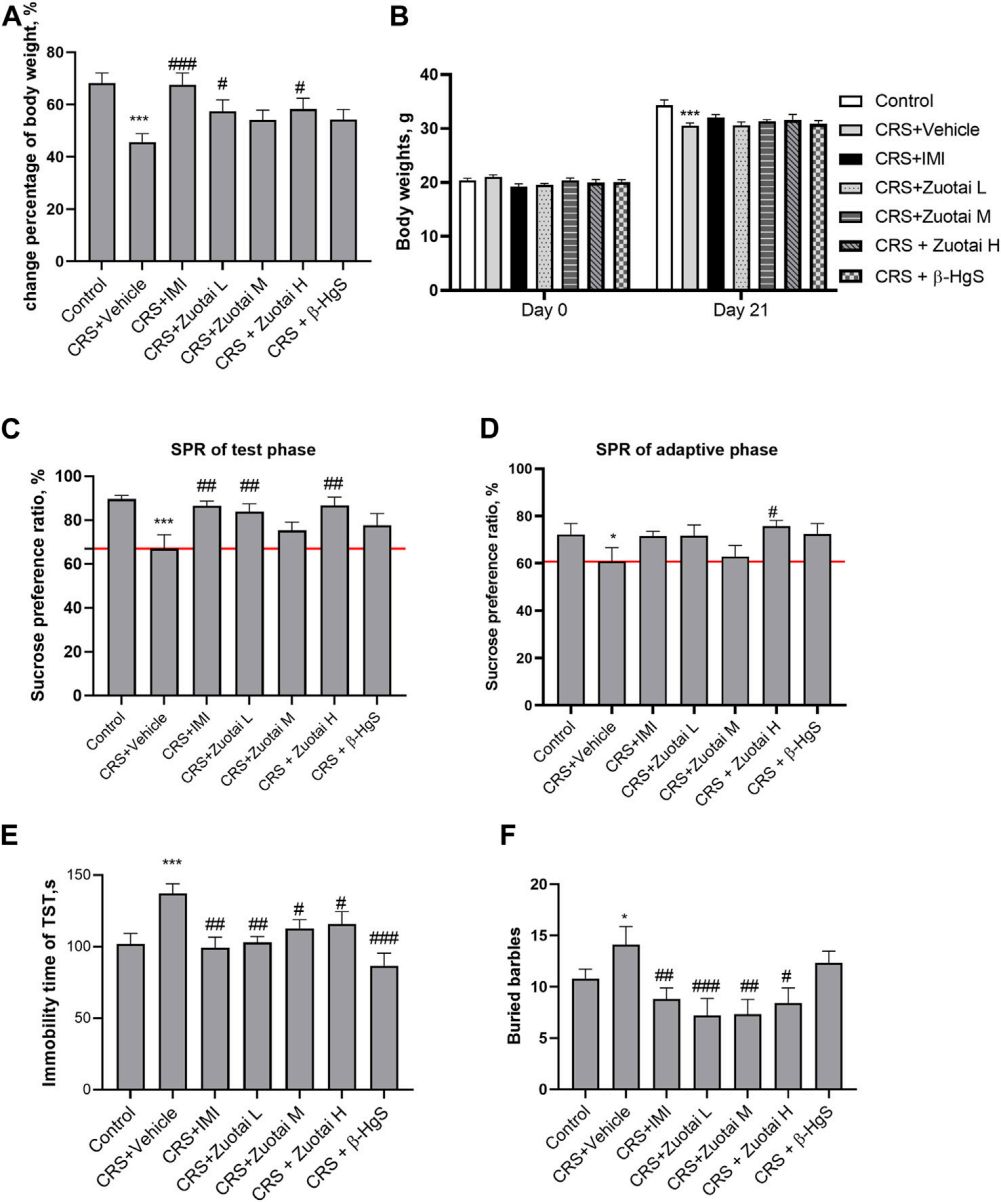
The effect of *Zuotai* and β-HgS on the depressive-like and anxious-like behaviors of chronic restraint-stressed (CRS) mice. **(A)** The percentage of body weight change between the beginning and at the end of mice experiment. **(B)** The body weights of mice at the beginning and at the end of mice experiment. **(C)** The sucrose preference ratio of mice during test phase. **(D)** The sucrose preference of mice during the adaption period of sucrose water. **(E)** The immobility time (behavioral despair state) of mice in suspension tail test (TST). **(F)** The buried marbles of mice in marble-burying test. Note: One-way ANOVA with Fisher’s Least Significant Difference (LSD) *post hoc* test was used; * means compared with control group, *p < 0.05, **p < 0.01, ***p < 0.001; # means compared with CRS + Vehicle group, #*p* < 0.05, ##*p* < 0.01, ## #*p* < 0.001; n = 10 for each group. Abbreviations: CRS (chronic restraint stress), SPT (Sucrose preference test), SPR (Sucrose preference ratio), TST (Tail suspension test), *Zuotai* L (*Zuotai* low dose), *Zuotai* M (*Zuotai* middle dose), *Zuotai* H (*Zuotai* high dose), IMI (imipramine).

In patients with depression, there are symptoms such as loss of appetite or hunger, and an increase or loss of weight (Merckmanuals, 2022). As shown in Figures 5A, B, CRS stress caused a significant decrease in body weight growth in chronic restraint stress (CRS) model group, and the body loss was reversed by the administration of *Zuotai* or β-HgS. The sucrose preference test was used to evaluate anhedonia, a typical symptom of depression, to judge the responses of experimental animals to rewards (Dang et al., 2009). As shown in Figure 5C, CRS significantly reduced sucrose preference in mice (*p* < 0.001), and *Zuotai* and β-HgS reversed the decreased sucrose preferences, especially in the CRS + *Zuotai* L (*p* < 0.01) and CRS + *Zuotai* H groups (*p* < 0.01). Even in the sucrose adaptation training stage, *Zuotai* and β-HgS were shown to reverse the reduced rate of sucrose preference induced by CRS (Figure 5D). The behavioral despair state of experimental animals is another key symptom similar to that of depression patients, which can be tested using the tail suspension test (Steru et al., 1985). As shown in Figure 5E, CRS significantly increased the immobility time (behavioral despair state) of the mice in the tail suspension test, while *Zuotai* significantly alleviated the behavioral despair state (CRS + *Zuotai* L < 0.01, CRS + *Zuotai* M < 0.05, CRS + *Zuotai* H < 0.05). In addition, β-HgS was also found to significantly reverse the immobility time (*p* < 0.001).

Patients with depression are often accompanied by an anxious mood (Merckmanuals, 2022). Marble burying test is a classic method used to evaluate the anxiety or repetitive stereotyped behavior of animals. Rodents have an innate tendency to bury objects. In the marble burying test, the more beads buried by a mouse, the more serious the mouse’s anxiety or obsessive-compulsive state (Deacon, 2009). As shown in Figure 5F, compared with the control group, the number of buried beads in the CRS model group increased significantly (*p* < 0.05). Compared with the model group, the CRS + *Zuotai* L (*p* < 0.001), CRS + *Zuotai* M (*p* < 0.05), CRS + *Zuotai* H (*p* < 0.05), and CRS + IMI groups (*p* < 0.01) all significantly decreased the number of buried beads.

These results suggested that *Zuotai* and β-HgS reversed CRS-induced depressive-like symptoms in mice including anhedonia, behavioral despair, and repetitive stereotyped behaviors.

### 3.2 *Zuotai* improved the behavioral performance of CRS mice in open field test

**FIGURE 6:**
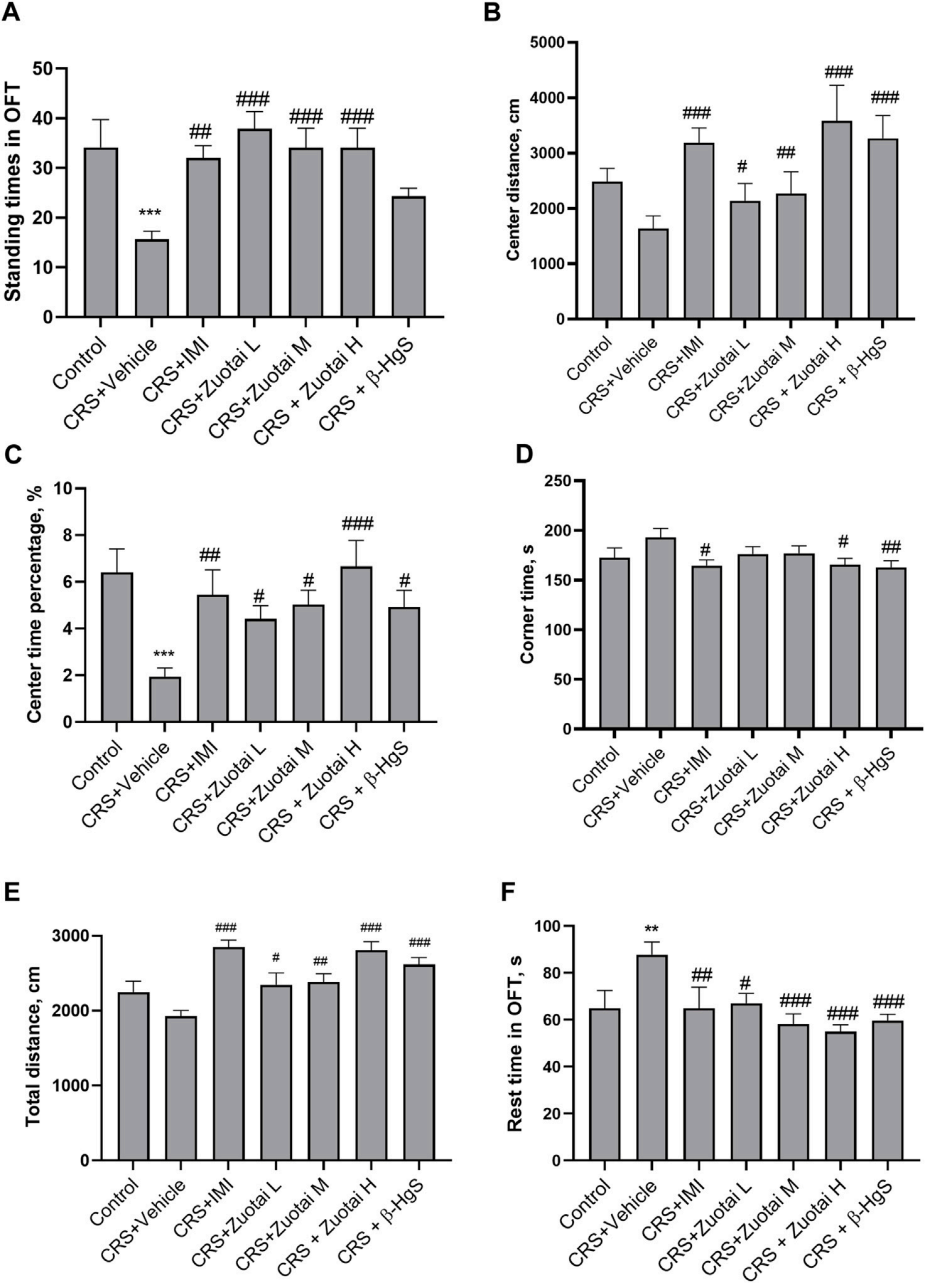
Effect of *Zuotai* and β-HgS on the behaviors of mice in open field test (OFT) at day 24. **(A)** The standing times of mice in OFT. **(B)** The movement distance of mice in central area in OFT. **(C)** Percentage of time mice spent in the central zone in OFT. **(D)** The rest time of mice in the four corners area in the opening experiment. **(E)** The total movement distance of mice in OFT. **(F)** The total rest time of mice in OFT. Note: One-way ANOVA with Fisher’s Least Significant Difference (LSD) *post hoc* test was used; * means compared with control group, *p < 0.05, **p < 0.01, ***p < 0.001; # means compared with CRS + Vehicle group, #*p* < 0.05, ##*p* < 0.01, ## #*p* < 0.001; n = 10 for each group. Abbreviations: CRS (chronic restraint stressed), OFT (Open field test), *Zuotai* L (*Zuotai* low dose), *Zuotai* M (*Zuotai* middle dose), *Zuotai* H (*Zuotai* high dose), IMI (imipramine).

The open field test is used to study the neuropsychiatric changes and various behaviors of experimental animals (Choleris et al., 2001; Wang et al., 2011). The standing number and activity of animals in the central area of open field are often considered to be indicators for exploring a novel environment (Walsh and Cummins, 1976; Choleris et al., 2001; Kalueff and Tuohimaa, 2004; Wang et al., 2011). As shown in Figures 6A–C, CRS significantly reduced the standing times and movement distance in the central area, as well as percentage of the time staying in the central area of mice. Both *Zuotai* and β-HgS reversed the reduction in the exploratory behaviors of mice, and showed a positive correlation between activity in the central area and administered *Zuotai* dose. The time that animals stayed in corners of the open field was correlated with the depression-like avoidance behavior of animals. It was found that CRS increased the total time of mice in corners, although there was no statistically significant difference. Compared with the CRS + Vehicle group, the rest time in the corners in the CRS + *Zuotai* H and CRS+β-HgS groups were significantly reduced (*p* < 0.05 and *p* < 0.01, respectively) (Figure 6D). These results showed that *Zuotai* and β-HgS alleviated the avoidance behaviors of CRS mice, and improved their exploratory behavior to new surroundings.

The total movement distance and the total rest time reflect the locomotor ability of mice (Walsh and Cummins, 1976). CRS reduced the movement distance of mice; however, the difference was not statistically significant (Figures 6E). Three doses of *Zuotai* significantly reversed the reduction of spontaneous activity in the mice caused by CRS in a dose-dependent effect relationship, and β-HgS also showed a similar effect. Correspondingly, CRS significantly increased the rest time of the mice in the open field test, while *Zuotai* and β-HgS significantly alleviated this effect of CRS (Figures 6F). That suggests that *Zuotai* and β-HgS improved locomotor tendency of mice.

### 3.3 *Zuotai* reversed the elevated stress hormone level in depressive mice

**FIGURE 7:**
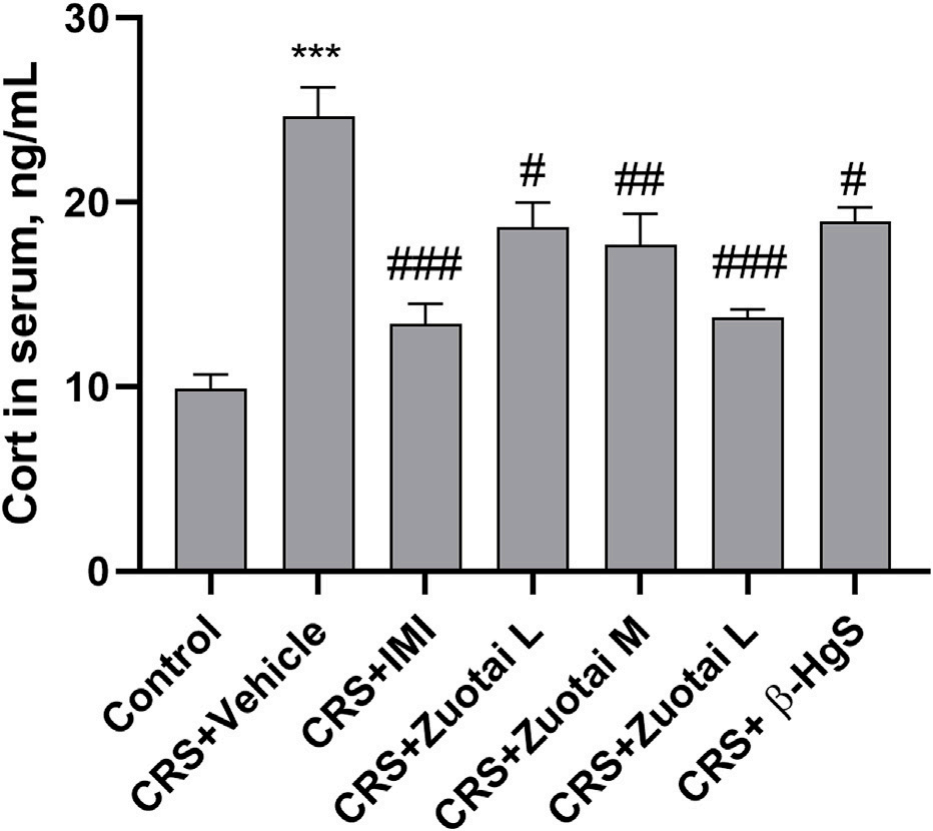
The *Zuotai* and β-HgS reversed the increased corticosterone (Cort) in CRS mice serum (n = 8, mean ± SEM). Note: One-way ANOVA with Tukey’s multiple comparisons test was used; * means CRS + Vehicle group was compared with control group, *p < 0.05, **p < 0.01, ***p < 0.001; # means compared with CRS + Vehicle group, #*p* < 0.05, ##*p* < 0.01, ## #*p* < 0.001; n = 8 for each group. Abbreviations: CRS (chronic restraint stress), Cort (corticosterone), *Zuotai* L (*Zuotai* low dose), *Zuotai* M (*Zuotai* middle dose), *Zuotai* H (*Zuotai* high dose), IMI (imipramine).

Glucocorticoid, cortisol for human or corticosterone (Cort) for mouse, in the blood is the commonly used hormone to assess stress level, and a key biomarker of the HPA axis, which has a direct correlation with the onset and development of depression (Nandam et al., 2020a). Clinical studies have reported that increased levels of cortisol in the blood of depression patients are associated with increased severity of depression and more suicide attempts (Nandam et al., 2020b; Nandam et al., 2020a; Choi et al., 2022). Significant Cort increases in the blood were also observed in various animal models of depression, such as chronic restraint stress, chronic unpredictable mild stress (CUMS), chronic social defeat (CSD), et al. (Planchez et al., 2019b; Nandam et al., 2020c). Therefore, this study used Cort to assess the effect of *Zuotai* on the stress level of CRS mice. The results showed that chronic restraint stress could significantly increase the serum Cort level of mice, and the anti-depression positive drug imipramine (IMI) sharply reversed this increase. Compared with the CRS + Vehicle group, the Cort levels in the mice serum of CRS + *Zuotai* L group, CRS + *Zuotai* M group, and CRS + *Zuotai* H group were significantly decreased (*p* < 0.05, *p* < 0.01, *p* < 0.001, respectively), with a positive dose-effect relationship. Furthermore, we found that β-HgS was also able to reduce the serum Cort increase induced by chronic restraint stress under the same pure HgS dose as in CRS + *Zuotai* L group (*p* < 0.05). The result is shown in Figure 7. The above indicates that *Zuotai* and β-HgS has potential to reduce the stress hormone level induced by chronic restraint stress in mice.

### 3.4 *Zuotai* decreased the periphery and CNS inflammation of depressive mice

**FIGURE 8:**
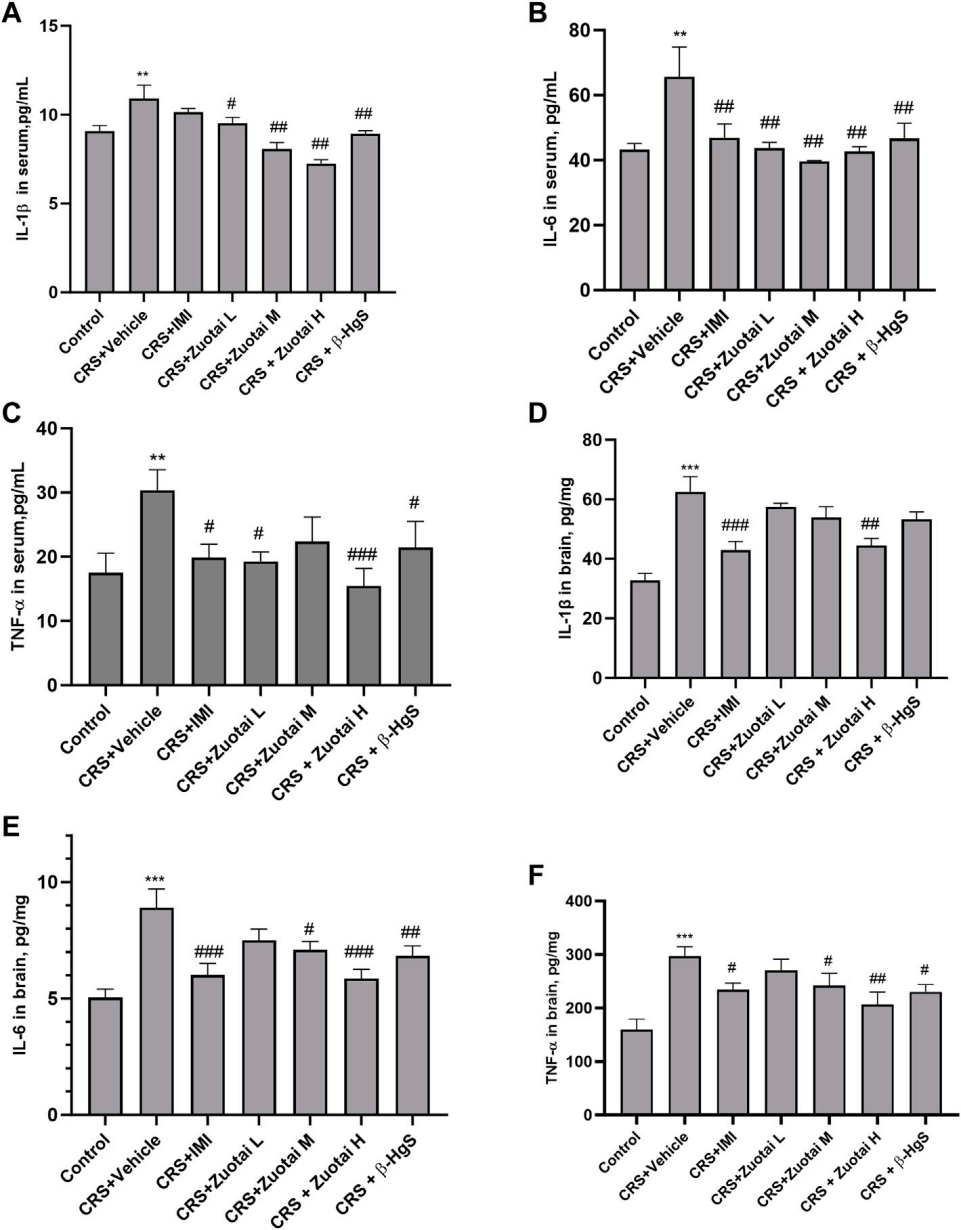
*Zuotai* and β-HgS reduced the periphery and CNS inflammation of CRS mice **(A)** The concentration of IL-1β in mice serum at day 26 (n = 10, Mean ± SEM). **(B)** The concentration of IL-6 in mice serum at day 26 (n = 10, Mean ± SEM). **(C)** The concentration of TNF-α in mice serum at day 26 (n = 10, Mean ± SEM). **(D)** The concentration of IL-1β in mice brain at day 22 (n = 8, Mean ± SEM). **(E)** The concentration of TNF-α in mice serum brain at day 22 (n = 8, Mean ± SEM). **(F)** The concentration of TNF-α in mice serum brain at day 22 (n = 8, Mean ± SEM). Note: One-way ANOVA with Fisher’s Least Significant Difference (LSD) *post hoc* test was used; * means CRS + Vehicle group was compared with control group, *p < 0.05, **p < 0.01, ***p < 0.001; # means compared with CRS + Vehicle group, #*p* < 0.05, ##*p* < 0.01, ## #*p* < 0.001; n = 10 for each group. Abbreviations: CRS (chronic restraint stress), IL-1β(interleukin-1β), IL-6 (interleukin-6), tumor necrosis factor-α (TNF-α), *Zuotai* L (*Zuotai* low dose), *Zuotai* M (*Zuotai* middle dose), *Zuotai* H (*Zuotai* high dose), IMI (imipramine).

The development of depression is often accompanied by an increased inflammatory response, which increases levels of peripheral and central pro-inflammatory cytokines, including interleukin-1β (IL-1β), interleukin-6 (IL-6), and tumor necrosis factor-α (TNF-α). This phenomenon has been observed in both clinical patients and animal models of depression, while antidepressant treatment can reduce the activated immune response in patients and model animals (Nesttis, 2021). Though the organ coefficient of the secondary immune organ, spleen, did not show a significant difference between the groups (*p* > 0.05, Figure 10J), we found that CRS significantly increased the levels of pro-inflammatory cytokines, IL-1β, IL-6, and TNF-α, in the blood (all, *p* < 0.01) and brains (all, *p* < 0.001) of mice, and the positive drug imipramine can significantly reverse the surge of these cytokines in blood and brains (Figures 8A–F). Importantly, *Zuotai* not only decreased the levels of these cytokines in blood and brains, but also showed a positive correlation of dose-effect relationship (Figures 8A–F). Moreover, β-HgS also decreased these cytokines in the blood and brains of CRS mice under the dose in this experiment (Figures 8A–F). Besides, some studies have reported that cytokines enter the brain through the leaky areas of ​​the blood-brain barrier, and directly act on the metabolism of monoamine neurotransmitters, thereby affecting consciousness and behavioral activities (Nesttis, 2021; Roohi et al., 2021). This indicates that the achieving antidepressant effect in the CRS mice of *Zuotai* and β-HgS may involve in down-regulating the inflammation in periphery and central neurological system (CNS).

### 3.5 *Zuotai* increased the levels of neurotransmitters and BDNF in depressive mice

**FIGURE 9:**
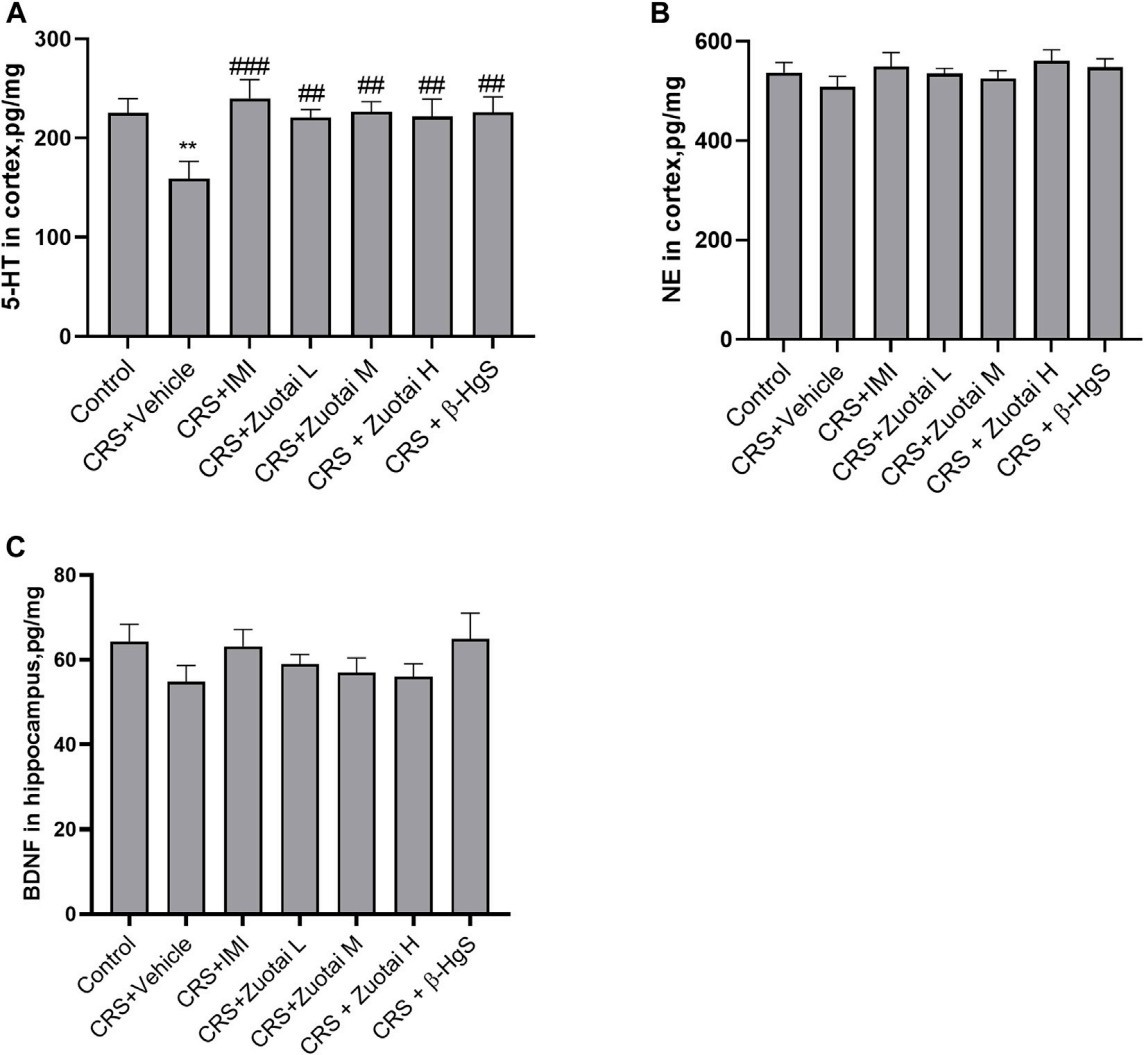
Influence of *Zuotai* and β-HgS on the monoamine neurotransmitters in brain cortex and the brain-derived neurotrophic factor (BDNF). **(A)** The concentration of serotonin (5-HT) in mice’s brain cortex at day 26. **(B)** The concentration of norepinephrine (NE) in mice’s brain cortex. **(C)** The concentration of brain-derived neurotrophic factor (BDNF) in mice’s brain hippocampus. Note: One-way ANOVA with Fisher’s Least Significant Difference (LSD) *post hoc* test was used; * means compared with control group, *p < 0.05, **p < 0.01, ***p < 0.001; # means compared with CRS + Vehicle group, #*p* < 0.05, ##*p* < 0.01, ## #*p* < 0.001; n = 10 for each group. Abbreviations: CRS (chronic restraint stress), 5-HT (erotonin), NE (norepinephrine), BDNF (brain-derived neurotrophic factor), *Zuotai* L (*Zuotai* low dose), *Zuotai* M (*Zuotai* middle dose), *Zuotai* H (*Zuotai* high dose), IMI (imipramine).

Depression is related to the lack or dysfunction of monoamine neurotransmitters in the central nervous system. Monoamine oxidase (MAO) inhibitors, tricyclics, 5-HT reuptake inhibitors and norepinephrine reuptake inhibitors can correct such dysfunctions (Elhwuegi, 2004). Due to depression poorly responding to potent dopamine agonists (Roohi et al., 2021), researchers pay more attention to the other two monoamines, serotonin (5-HT) and norepinephrine (NE) to find antidepression therapeutics. So, in this study, we investigated the effects of *Zuotai* on the two monoamine neurotransmitters (5-HT and NE) in the central nervous system of CRS mice. We found that CRS significantly reduced the content of 5-HT in the cerebral cortex of CRS mice, while imipramine, *Zuotai,* and β-HgS increased the levels of 5-HT (Figure 9A). We also found that *Zuotai* and β-HgS had similar boosting effects on another neurotransmitter, NE, which is involved in the development of depression, although this effect was not statistically significant (Figure 9B). Together, these results suggested that *Zuotai* and β-HgS reversed the reduction of monoamine neurotransmitters in the central nervous system caused by CRS.

BDNF is a brain-derived trophic factor that is important for the survival and growth of neurons, as well as in maintaining synaptic plasticity. There are high levels of BDNF in the hippocampus, cerebral cortex, and basal forebrain region, which are critical for learning and memory. BDNF was found to decrease in the brains of both depression patients and depressive-model animals (Yang et al., 2020). Our study found that CRS decreased the levels of BDNF in the hippocampus, and the administration of imipramine, *Zuotai* and β-HgS slightly reversed this decrease, although the results were not statistically significant (*p* > 0.05) (Figure 9C).

### 3.6 The accumulation of mercury from *Zuotai* in the organs of CRS mice

**FIGURE 10:**
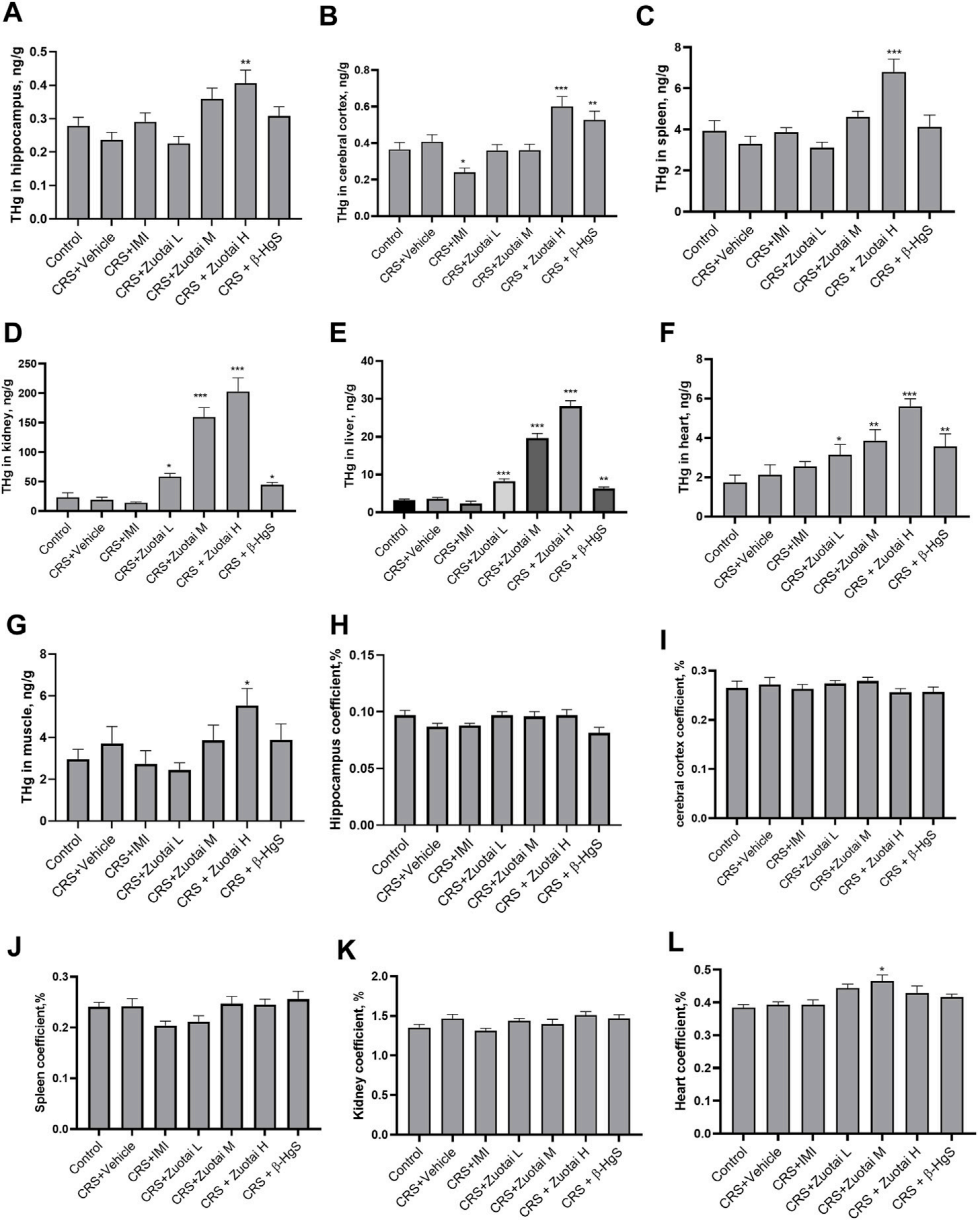
The risk assessment of Hg from *Zuotai* and β-HgS on the brain, kidney, and other organs. **(A–G)**: The total mercury (THg) concentration of hippocampus (n = 8), brain cortex (n = 8), spleen (n = 10), kidney (n = 10), liver (n = 10), heart (n = 10), and muscle (n = 10). **(H–L)**: The organ coefficients of hippocampus (n = 8), brain cortex (n = 8), kidney (n = 10), heart (n = 10), and spleen (n = 10). Note: One-way ANOVA with Fisher’s Least Significant Difference (LSD) *post hoc* test was used; * means compared with control group, *p < 0.05, **p < 0.01, ***p < 0.001; n = 10 for each group. Abbreviations: CRS (chronic restraint stress), THg (total mercury), *Zuotai* L (*Zuotai* low dose), *Zuotai* M (*Zuotai* middle dose), *Zuotai* H (*Zuotai* high dose), IMI (imipramine).

Although *Zuotai* showed antidepressant activity in the CRS depression mouse model, the drug contains approximately 54.5% β-HgS (Wang et al., 2010a; Li et al., 2015a), which raises concerns about the risk of mercury accumulation in body. It is well known that mercury is a toxic heavy metal ubiquitous in nature. Different chemical forms of mercury have large differences in toxicity. It is generally agreed that the toxicity of mercury has the following order: methylmercury > mercury vapor (vapor Hg^0^) > soluble inorganic mercuric compounds >> liquid mercury (liquid Hg^0^) >> mercuric sulfide (Liu et al., 2018). β-HgS has very low solubility product constant (*K*_*sp*_), 1.8 × 10^−53^ (Dean, 1999), and mercury in it is difficult to be absorbed by digestive tract (EPA, 2002). Our study found that continuous administration of *Zuotai* for 21 days increased the concentrations of total mercury (THg) in the hippocampus, cortex, spleen, kidney, liver, heart, and muscle of mice, especially in the kidney, and showed strong dose-dependent effects (Figures 10A–G). The mean mercury concentrations of the hippocampus, cortex, spleen, kidney, liver, heart, and muscle in CRS + *Zuotai* H group were 0.406 ± 0.124, 0.601 ± 0.172, 6.79 ± 2.01, 202.88 ± 73.577, 28.05 ± 4.59, 5.6 ± 1.21, and 5.54 ± 2.551 ng/g, respectively. In addition, the organ coefficient is an important indicator used to reflect drug toxicity or side effect. Compared with the control group, the organ coefficients (hippocampus, cerebral cortex, spleen, kidney, liver) of mice in each test group showed no significant change, except for the increase of heart coefficients of the *Zuotai* M-treated group mice (*p* < 0.05). It was speculated that the abnormal increase in the heart coefficient in this group was due to the failure to remove the remaining coagulated blood clots in the heart during weighing, but this needs to be verified in the future. The above shows the *Zuotai* and β-HgS can cause a certain extent of increase in main organs under the doses in this experiment for 21 days consecutive oral administration.

### 3.7 *Zuotai* has no observed adverse effect on the H&E staining histology of hippocampus

**FIGURE 11:**
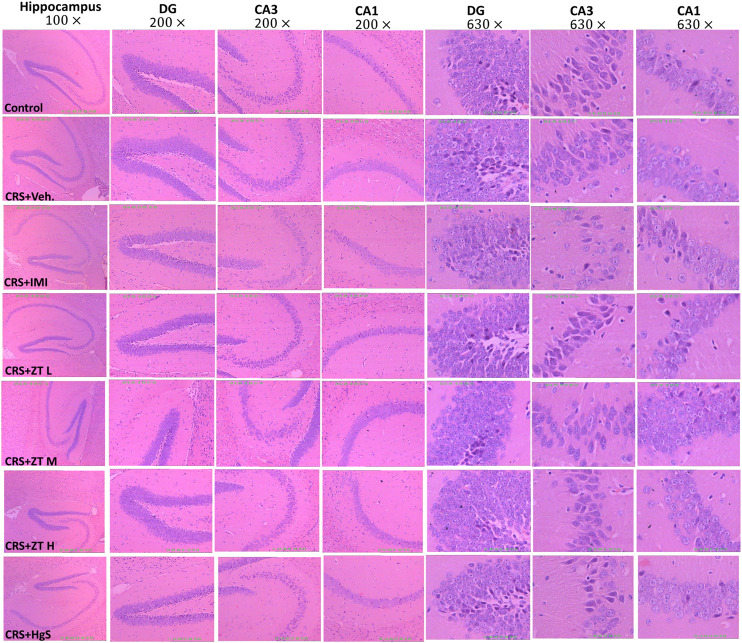
Influence of *Zuotai* on the H&E staining histology of mice hippocampus. Two mice were selected from each group, and whole brains were collected and fixed with 4% paraformaldehyde at day 26, and stained with H&E. The pathology of the hippocampus was observed under 10 × , 20 × , and 63 × magnification with a light microscope. Abbreviations: CRS (chronic restraint stress), DG (dentate gyrus), CA1(Cornu Ammonis 1 region), CA3 (Cornu Ammonis 3 region), *ZT* L (*Zuotai* low dose), *ZT* M (*Zuotai* middle dose), *ZT*H (*Zuotai* high dose), IMI (imipramine), Veh. (vehicle).

The patients with major depression disorder often had atrophy of hippocampus, accompanying changes in neuronal plasticity (Espinoza Oyarce et al., 2020; Yang et al., 2020), and a few studies has found that CRS caused some changes in mice hippocampus (Yun et al., 2010; Huang et al., 2015). It has been sufficiently verified that the high level of mercury exposure (especially vapor Hg^0^ and organic mercury) can cause damage to the central nervous system, including hippocampus (EPA US, 1997; WHO, 2008; ATSDR, 2022). In this study, we have found that *Zuotai* and β-HgS could cause a certain extent of Hg increase in hippocampus, which probably posed a risk to cause pathological injury. Therefore, here we examined hippocampus histology by using H&E staining. The result shows that there were no obvious morphology changes in the CRS mice hippocampus, compared with the control group (Figure 11), which is distinct from the literature reported (Yun et al., 2010; Huang et al., 2015). Compared with control and CRS groups, *Zuotai* and β-HgS did not cause markedly pathological changes in mice hippocampal (Figure 11). The histological characteristics in all groups are the following: the dentate gyrus (DG), CA1, and CA3 of hippocampus had a clear contour; neurons were arranged regularly, and pyramidal cells and granule cells had normal sizes; the granule cells were slightly increased in some areas, but cell morphology had no obvious abnormality; there was no interstitial edema, and some small blood vessels were dilated; vascular endothelial cells did not show proliferation (Figure 11). Besides, the organ coefficient of mice hippocampus in the CRS + Vehicle group decreased slightly when compared with the control group, and that was mildly reversed by *Zuotai*, although the results were not statistically significant (*p* > 0.05) (Figure 10H). Therefore, *Zuotai* has no observed adverse effect on the H&E staining histology of hippocampus and the hippocampus coefficient.

### 3.8 *Zuotai* has no obvious effect on the decreased newborn cells in dentate gyrus caused by chronic restraint stress

**FIGURE 12:**
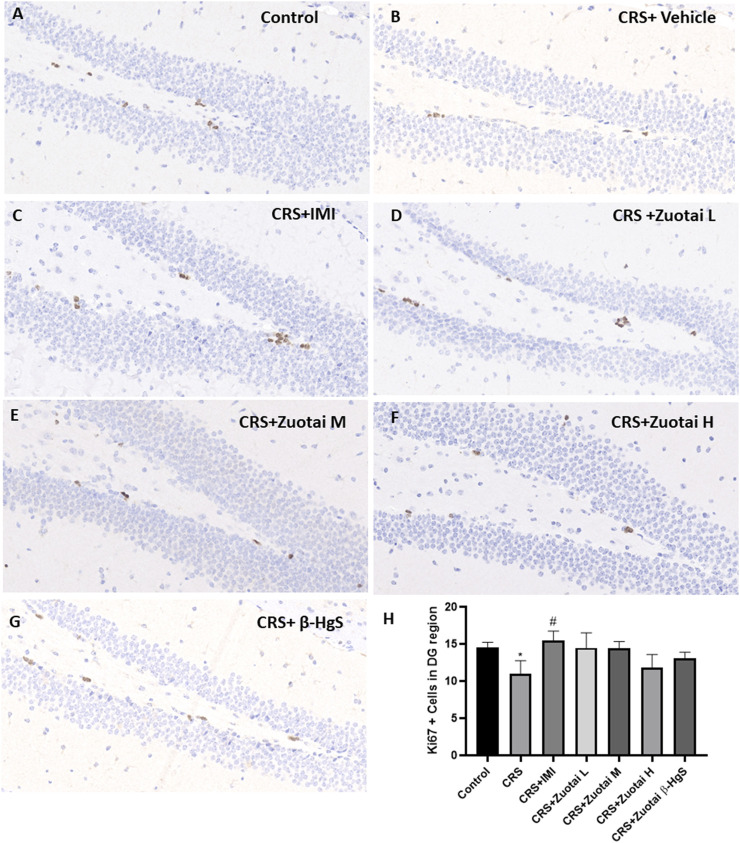
Influence of *Zuotai* on the Ki67 (cell proliferation marker) positive cells in mice dentate gyrus (DG) at day 22. **(A–G)** are the Ki67 IHC staining in the dentate gyrus of Control, CRS + Vehicle, CRS + IMI, CRS + *Zuotai* L, CRS + *Zuotai* M, CRS + *Zuotai* H groups mice. **(H)** The Ki67 positive cells number in dentate gyrus (4 mice each groups, Mean ± SD). The whole brains were selected from four mice each group at day 22, then fixed with 4% paraformaldehyde. The ICH staining has been done for cell proliferation marker Ki67 and was observed under 40 × magnification with a light microscope. Note: One-way ANOVA with Fisher’s Least Significant Difference (LSD) *post hoc* test was used; * means CRS + Vehicle group was compared with control group; # means compared with CRS + Vehicle group, #*p* < 0.05. Abbreviations: CRS (chronic restraint stress), DG (dentate gyrus), *Zuotai* L (*Zuotai* low dose), *Zuotai* M (*Zuotai* middle dose), *Zuotai* H (*Zuotai* high dose), IMI (imipramine).

The dentate gyrus subgranular zone (SGZ) of the hippocampus is one of the few brain regions in adult mammals, including human, that have the ability to regenerate neurons throughout life (Zamproni et al., 2021). The neuronal regeneration in dentate gyrus is sensitive to a variety of environmental factors and stimulus factors (Kuhn et al., 1996; Kempermann et al., 1997; Gould et al., 1998; Van Praag et al., 1999). And, experimental stress (physical or psychological) has been shown to quantitatively inhibit the formation of new cells in the mammalian dentate gyrus (Gould et al., 1997; Gould et al., 1998; Tanapat et al., 1998), and both acute and chronic stress treatment procedures also decreased cell proliferation (Fuchs et al., 1997). As a classic biomarker for detecting cell proliferation ability in tissues, Ki67 was used to explore the effect of *Zuotai* on the neuronal regeneration of dentate gyrus. It was found that compared with the control group, the number of Ki67 positive cells in the dentate gyrus of CRS + Vehicle group mice was significantly reduced (*p* < 0.05), while other groups (three *Zuota*i-treated groups, CRS+β-HgS group and CRS + IMI group) did not show a significant increase (*p* < 0.05). Then, compared with the CRS + Vehicle group, CRS + IMI group had a significant increase of Ki67 cells in the dentate gyrus (*p* < 0.05), and although the three *Zuota*i-treated groups (with order of CRS + *Zuotai* L group > CRS + *Zuotai* M group> CRS + *Zuotai* H group) and CRS+β-HgS group also increased the number of Ki67 positive cells in the dentate gyrus to a certain extent, there were no statistical difference (*p* > 0.05). Therefore, *Zuotai* and β-HgS show no obvious effects on the cell proliferation of the dentate gyrus in chronic restraint stress mice. The result is shown in Figure 12.

## 4 Discussion

*Zuotai* is a classic mineral-herbal mixture of Tibetan medicine containing β-HgS, which is often used to prepare compound preparations for treating of nervous system diseases. In view of the unmet demand for the treatment of depression, identifying new antidepressants from traditional medicines is an important approach. Our previous study has reported that *Zuotai* alleviated depression-like behaviors in chronic unpredictable mild stress (CUMS) model (Zhao et al., 2018). Present study used another depression model, chronic restraint stress model (CRS), to further validate the previously reported findings. Through behavioral testing, we found that mineral-herbal mixture *Zuotai* containing β-HgS and β-HgS ameliorated depression-like behaviors in the model mice, such as anhedonia, behavioral despair state, and anxiety, as well as improving results of exploratory behaviors in open field test. The elevation of pro-inflammatory factors (IL-1β, IL-6, and TNF-α) caused by CRS was reduced, and the decrease of 5-HT in the cerebral cortex and BDNF in the hippocampus induced by CRS was also increased. In addition, *Zuotai* and β-HgS were found to increase the mercury concentration in the hippocampus, cortex, spleen, kidney, liver, heart, and muscle with a positive dose-effect relationship; however, histopathological examinations did not find a significant difference in the hippocampus between control and treated animals. The organ coefficients of hippocampus, kidney, spleen, and other organs also showed no significant difference.

Depression Patients have the symptoms of persistent low mood, anhedonia, despair, and helplessness, slow thinking or action, and anxiety (Bentley et al., 2014; Merckmanuals, 2022), and are often accompanied by low functioning of monoamine neurotransmitters in the central nervous system (Elhwuegi, 2004). Many animal models have been developed to simulate clinical symptoms of depression. For example, chronic restraint stress model animals display anhedonia, behavioral despair, reduced exploring curiosity, repetitive stereotyped activities, and decreased spontaneous movements, and anti-depressive drugs can reverse these changes (Ulloa et al., 2010; Chiba et al., 2012; Ding et al., 2016). In this study, we treated chronic restraint-stressed mice with *Zuotai* or β-HgS, and these behaviors in the model mice were reversed. And, similar antidepression-like behaviors effects of *Zuotai*, β-HgS and cinnabar also were observed in CUMS model mice (Zhao et al., 2018), CRS + CUMS model mice (Qiao et al., 2022), and normal mice (Dai et al., 2014), respectively.

The insufficiency of monoamine neurotransmitters plays an important role in the pathogenesis of depression. 5-HT has the effect of improving mood, and increasing enthusiasm. It is mainly synthesized by 5-HT neurons in the dorsal raphe nucleus, and then projected to certain brain regions including the cerebral cortex, hippocampus, amygdala, and striatum (Frazer and Hensler, 1999). Deficiency of 5-HT results in low mood, anxiety, and lack of motivation and willfulness (Mohammad-Zadeh et al., 2008; Celada et al., 2013; Ślifirski et al., 2021). We here found that *Zuotai* and β-HgS reversed the reduction of 5-HT in the brain cortex of CRS model mice. Our previous study also found that *Zuotai* reversed the reduced 5-HT level in the brain cortex of CUMS model mice (Zhao et al., 2018), and β-HgS enhanced the brain cortex 5-HT increase induced by antidepressant (sertraline) in the CRS + CUMS model mice (Qiao et al., 2022). However, there also have reports that cinnabar (α-HgS) suppressed 5-HT levels in the whole brain of normal C56BL/6 mice (Wang et al., 2007). We speculated that the possible reasons for this difference are the health or disease status of mice, drug dose, the distribution heterogeneity of 5-HT in brain, mice strain, or others. Besides, norepinephrine (NE) is another important psycho-emotional monoamine neurotransmitter, which is mainly synthesized by NE neurons in the locus coeruleus of the brainstem, and projected to the cerebral cortex, limbic forebrain, and hypothalamus through fibers of the ascending projection system (Benarroch, 2009; Ross and van Bockstaele, 2021). In this study, we found that the NE level had an increase tendency in the brain cortex of *Zuotai* and β-HgS group mice compared with CRS group, but there is no statistical significance. This conflicts with our previous study based on the CUMS model in KM mouse (Zhao et al., 2018). We previously found that *Zuotai* reversed the reduced levels of NE in the cortex in CUMS mice (Zhao et al., 2018), and β-HgS had same effect in CRS + CUMS mice (Qiao et al., 2022). The reason for this difference may be due to the different backgrounds of NE in the two models, because previous study found that CUMS significantly induced the reduction of cortical NE in model mice (Zhao et al., 2018), while CRS was not found to reduce NE in the present study. In conclusion, the improvement of depression-like behaviors of the CRS model mice by *Zuotai* and β-HgS involved intervention of the central monoamine neurotransmitter system, including that of 5-HT.

The strong link between inflammation and depression has been confirmed by a growing body of evidence. Clinically, a considerable number of depression patients, especially those with treatment-resistant depression, not only have significantly increased peripheral inflammatory response markers (Nesttis, 2021; Beckett and Niklison-Chirou, 2022), but also exhibit activated inflammation states in the brain as confirmed by imaging examinations (Han and Ham, 2021). Additionally, all cytokines, brain trauma, certain vaccines, and exogenous immune toxins can cause depression-like symptoms in both humans and experimental animals (Felger and Lotrich, 2013; Bodnar et al., 2018). Moreover, CUMS, CRS, and chronic social defeat stress (CSDS) can also trigger activation of peripheral and central immunities in animals, and antidepressant intervention is able to reduce the inflammation level (Han and Ham, 2021; Kitaoka, 2022; Wang et al., 2022). Previous studies have reported that inflammatory responses imposed an important impact on the brain and behavior, especially cortical neural circuits and neurotransmitter systems related to motivation, motor ability, anxiety, interest arousal, and vigilance (Miller et al., 2013). Increased peripheral blood inflammatory markers are associated with a connectivity decrease of reward circuits between ventral striatum and ventromedial prefrontal cortex (vmPFC), which in turn correlated with increased anhedonia (Felger et al., 2016). For the neurotransmitter system, inflammation not only increases the expression and function of presynaptic reuptake pumps for serotonin, dopamine, and norepinephrine, but also decreases tryptophan hydroxylase cofactors such as tetrahydro biopterin, thereby diverting tryptophan to synthesize kynurenine to reduce the synthesis monoamine transmitters (Miller et al., 2013; Suneson et al., 2021). Besides, inflammation-induced reduction of the synthesis and release of monoamine neurotransmitters (5-HT and dopamine) has been observed in humans and animals, and is directly associated with reduced motivation in animals (Miller et al., 2013; Felger and Treadway, 2017). Consistent with existing studies, we found that *Zuotai* and β-HgS reduced the levels of inflammatory markers (IL-1β, IL-6, and TNF-α) in CRS mice. Based on above, we hypothesize that the improvement of depressive-like behaviors by *Zuotai* and β-HgS probably also was achieved by decreasing the inflammation induced by CRS, thus alleviating the dysfunctions of neural circuits and the monoamine neurotransmitter system.

BDNF is a key regulator for neuronal plasticity in the brain, which has been increasingly associated with rapid antidepressant effects. Preclinical and clinical studies have reported that impaired BDNF signaling through its receptor TrkB in the pathophysiology of mood disorders (Castrén and Monteggia, 2021). BDNF expression decreased in the *postmortem* brain samples of depressed patients, especially in the hippocampus and amygdala, while antidepressant treatment increases BDNF in the brain (Dwivedi, 2013; Castrén and Monteggia, 2021). Stressed rodents had an abnormal/malfunctional state of synaptic plasticity in cortical brain regions (Patel et al., 2019). Ketamine exerted rapid antidepressant effects through improving BDNF-related synaptic plasticity in the hippocampus and prefrontal cortex (Castrén and Monteggia, 2021). In addition, inflammation is related to the reduction of BDNF in central nervous system. Specifically, inflammation can promote tryptophan production, the precursor of 5-HT, to synthesize kynurenine, which is then metabolized to quinolinic acid and leads to the increase of neurotransmitter glutamate. Excess glutamate has been proven to reduce BDNF and cause excitotoxicity (Castrén and Monteggia, 2021). We found that *Zuotai* and β-HgS decreased inflammation caused by CRS, and also increased BDNF in the hippocampus, although the results were not statistically significant (*p* > 0.05). This effect is also observed in other studies using CRS mice (Chiba et al., 2012) or CRS rats used as model animals (Chiba et al., 2012). Taken together, the anti-depressant-like behavioral effect of *Zuotai* and β-HgS may involve up-regulating inflammation-related BDNF in the hippocampus, which is directly associated with neuronal plasticity and stability.

In spite of the anti-depressive potential found in this study, and considering that *Zuotai* is a mineral-herbal mixture containing β-HgS, it is necessary to evaluate its risk of mercury exposure. The mercury toxicity is closely related to its chemical form. Organic mercury (such as MeHg) has the greatest toxicity; gaseous elemental mercury, soluble inorganic mercury, and liquid elemental mercury have less toxicity, and insoluble inorganic mercury such as β-HgS has the least toxicity (Liu et al., 2018). The US Environmental Protection Agency (EPA) and the World Health Organization (WHO) reported that the solubility of HgS is extremely low (*Ksp* of *β*-HgS is 1.6 × 10^−52^; *Ksp* of *α*-HgS is 4.0 × 10^−53^ (Dean, 1999) and difficult to be absorbed by the body (EPA, 2002; Registry, 2003), and its toxicity is very low (Liu et al., 2008; Liu et al., 2018; Liu et al., 2019). We found that *Zuotai* increased total Hg levels in the brain (cortex and hippocampus), kidney, liver, spleen, heart, and muscle in a dose-dependent manner; the Hg concentration in the kidney was the highest among organs and tissues, and the highest renal Hg concentration (202.88 ± 73.577 ng/g) was in *Zuotai* high-dose group (607 mg/kg, i.g. for 21 days). For inorganic mercuric salt, U.S. Department of Health and Human Services Agency for Toxic Substances and Disease Registry (ATSDR) reported that the kidney is the uppermost accumulation organ, with 40%–50% of the whole body burden of Hg, so kidney is extreme sensitive to inorganic mercuric salt; the following accumulated organ for inorganic mercuric salt is liver, with 10%–20% of the whole body burden of Hg (ATSDR, 2022). The EPA reported that the lowest observed adverse effect level (LOAEL) dose of mercuric chloride after 6-month oral administration in B6C3F1 mouse was 5 mg/kg day (namely, 3.7 Hg mg/kg day), and the corresponding total Hg concentrations in kidney and liver were 36,100–40,600 ng/g and 2,980–3,380 ng/g, respectively (National Toxicology Program, 1993; EPA US, 1997)*.* Yang et al. reported that the total Hg concentrations in the kidney of SD rats after 6-month *Zuotai* (dose 53.4 mg/kg day) administration were 252.4 ± 152.6 ng/g and no damage of kidney and liver was observed by the serum indicators of kidney function, H&E staining, and organ coefficient (Yang et al., 2004). In our study, the highest renal Hg concentration and liver Hg concentration in the CRS mice treated with high-dose *Zuotai* were 202.88 ± 73.58 ng/g and 28.05 ± 4.59 ng/g respectively, which were lower than that in the above reports. Besides, present study found *Zuotai* had no observed adverse influence on the H&E staining histopathology in brain, the cell proliferation ability in dentate gyrus, and the organ coefficients (hippocampus, cerebral cortex, kidney, liver, spleen) in CRS mice. The result is consistent with the reports that *Zuotai* or HgS toxicity were far less than others mercury chemical forms (Liu et al., 2016; Wu et al., 2016; Zhang et al., 2017). Summarily, the Hg concentration in organs from *Zuotai* at least in kidney and liver was still within the safe range under the doses and experimental duration used in this study, although *Zuotai* increased the concentration of Hg in these organs in a certain extent. And, *Zuotai* has not obviously observed adverse effect on brain under the same experimental condition.

There still are some limitations in this study. 1) We only tested monoamine transmitters in the cortex and BDNF in the hippocampus. Considering the spatial heterogeneity of these factors in the brain, further testing of these indicators, as well as their related receptors and synthetic or metabolic enzymes is warranted to better understand the antidepressant mechanism. 2) In this study, only H&E staining and Ki67 IHC staining were conducted, so it probably is more reliable if other staining methods (Nissl staining, Bielschowsky silver staining, IF, et al.) were applied. 3) It is known that neuroendocrine pathways, such as the HPA axis, have an important role in the pathogenesis of depression. Here we just assayed the stress hormone Cort for assessing the mice stress level, so how *Zuotai* impacts on the neuroendocrine metabolism is still unknown. 4) It has been found that tryptophan, the precursor of 5-HT synthesis, diverts to the kynurenine synthesis pathway, which mediates the effect of inflammation on neuropsychology and behavior. We just showed that *Zuotai* improved the inflammatory state of the CRS mice and upregulated 5-HT; however, the detailed regulatory mechanisms are still unclear.

In conclusion, *Zuotai* exhibited an effect on alleviating the depressive behaviors in chronic restraint stress (CRS) mouse model, accompanying with reversing the elevated stress hormone and the activated peripheral and cerebral inflammation, and increasing the monoamine neurotransmitter in brain. Although *Zuotai* increased the mercury amount in organs, the Hg concentration was still within the safe range, at least in kidney and liver; and *Zuotai* showed no obviously observed adverse effect on brain under this experimental condition. Finally, this study suggests the potential feasibility of repositioning traditional medicines, particularly mineral-herbal mixtures like *Zuotai*.

## Figures and Tables

**Table:** 

**CRS**	chronic restraint-stressed
**CSDS**	Chronic social defeat stress
**CUMS**	Chronic unpredictable mild stress
**IMI**	Imipramine
**HgS**	Mercuric sulfide
**5-HT**	Serotonin
**NE**	Norepinephrine
**IL-1β**	Interleukin 1 beta
**IL-6**	Interleukin 6
**TNF-α**	Tumor necrosis factor alpha
**BDNF**	Brain-derived neurotrophic factor
**Cort**	Corticosterone
**DG**	Dentate gyrus
**CA1**	Cornu Ammonis 1 region
**CA3**	Cornu Ammonis 3 region
**SGZ**	Subgranular zone of the dentate gyrus
**SVZ**	Subventricular zone
**SPT**	Sucrose preference test
**SPR**	Sucrose preference rate
**TST**	Tail suspension test
**OFT**	Open field test
**HPA axis**	Hypothalamic-pituitary-adrenal axis
**Ksp**	Solubility Product Constant
**EPA**	U.S. Environmental Protection Agency
**WHO**	World Health Organization
**ATSDR**	Agency for Toxic Substances and Disease Registry
**XRD**	X-ray diffraction; Elisa: Enzyme-linked immunosorbent assay
**KM mice**	Kunming mice
**IHC**	Immunohistochemistry
**SPF**	Specific pathogen-free
**Zuotai L**	Zuotai low dose
**Zuotai M**	Zuotai middle dose
**Zuotai H**	Zuotai high dose
**H&E**	Hematoxylin and eosin
**Total Hg or THg**	Total mercury content
**CNS**	Central neurological system
**MAO**	Monoamine oxidase
**One-way ANOVA**	one-way analysis of variance
**SD**	Standard deviation
**SEM**	Standard error
**LSD**	Least significant difference
**CAS**	Chinese Academy of Sciences

## Data Availability

The raw data supporting the conclusion of this article will be made available by the authors, without undue reservation.
